# Thermally Stable UV-Curable Pressure-Sensitive Adhesives Based on Silicon–Acrylate Telomers and Selected Adhesion Promoters

**DOI:** 10.3390/polym16152178

**Published:** 2024-07-30

**Authors:** Agnieszka Kowalczyk, Krzysztof Kowalczyk, Jan Gruszecki, Tomasz J. Idzik, Jacek G. Sośnicki

**Affiliations:** Department of Chemical Organic Technology and Polymeric Materials, Faculty of Chemical Technology and Engineering, West Pomeranian University of Technology in Szczecin, 70-322 Szczecin, Poland; kkowalczyk@zut.edu.pl (K.K.); gj50107@zut.edu.pl (J.G.); tomasz.idzik@zut.edu.pl (T.J.I.); jacek.sosnicki@zut.edu.pl (J.G.S.)

**Keywords:** photopolymerization, telomerization, adhesion promoter, pressure-sensitive adhesives, silicone, acrylate

## Abstract

A new type of UV-curable pressure-sensitive adhesive containing Si atoms (Si-PSAs) was prepared by a solution-free UV-initiated telomerization process of n-butyl acrylate, acrylic acid, methyl methacrylate, and 4-acrylooxybenzophenone using triethylsilane (TES) as a telogen and an acylphosphine oxide (APO) as a radical photoinitiator. Selected commercial adhesion promoters were tested as additives in the formulation of adhesive compositions, i.e., (i) an organic copolymer with polar groups (carboxyl and hydroxyl); (ii) a hydroxymetal-organic compound; and (iii) a quaternary ammonium salt and (iv) a chlorinated polyolefin. No fillers, crosslinking agents, or photoinitiators were used in the adhesive compositions. NMR techniques confirmed the incorporation of silicon atoms into the polyacrylate structure. The influence of adhesion promoters on the kinetics of the UV-crosslinking process of Si-PSAs was investigated by a photo-DSC technique. The obtained Si-PSAs were characterized by adhesion (to steel, glass, PMMA, and PE), tack, and cohesion at 20 °C. Finally, the wetting angle of Si-PSAs with water was checked and their thermal stability was proved (TGA). Unexpectedly, the quaternary ammonium salt had the most favorable effect on improving the thermal stability of Si-PSAs (302 °C) and adhesion to glass and PMMA. In contrast, Si-PSAs containing the hydroxymetal-organic compound showed excellent adhesion to steel.

## 1. Introduction

Pressure-sensitive adhesives (PSAs) are a special type of adhesives characterized by their ability to adhere to a variety of substrates when subjected to a slight pressure [1]. Adhesive binders of PSAs can be polyurethanes, polyacrylates, polyesters, polyethers, silicone resins, vinyl acetate copolymers, or rubber [2]. One of the most important classes comprises the polyacrylate pressure-sensitive adhesives, largely due to the low glass transition temperature values (T_g_) of these (co)polymers, making them used industrially as single- and double-sided adhesive tapes, labels, or protective films. Polyacrylate PSAs are transparent, colorless, and resistant to aging under sunlight, but they are not thermally resistant [3,4,5]; in the case of exposure of adhesive joints to high temperatures, pressure-sensitive silicone adhesives perform well. The unique combination of properties of silicones, such as high flexibility of the Si-O-Si backbone, negligible intermolecular interactions, low surface tension, excellent thermal stability, and high UV transparency, support the fact that silicone PSAs perform well at extremely high and low temperatures, and they have excellent electrical, chemical and weathering properties compared to classical organic PSAs [6,7,8].

Silicone PSAs consist of two main components: a silicone polymer and a resin. The silicone polymer is usually poly(dimethylsiloxane) or high-molecular-weight poly(dimethyl/diphenylsiloxane), which contains a residual silanol group (SiOH) at the ends of the chains. On the other hand, silicone resin (named MQ) is a three-dimensional silicate structure consisting of trimethylsiloxane groups and silsesquioxane units. The MQ resin is synthesized from sodium silicate and chlorosilane through hydrolysis and condensation reactions in a multi-step process. It is supplied in a hydrocarbon solvent such as toluene or xylene. Neither the silicone polymer nor the MQ resin alone exhibits adhesive properties. These features are observed only for their mixture in the appropriate weight ratio; a crosslinking process is required to transform them into PSAs as well. This process can take place in two ways, i.e., by peroxide-initiated free-radical crosslinking or platinum-catalyzed addition crosslinking. In the former, the organic peroxides (e.g., 2,4-dichlorobenzoyl peroxide and dibenzoyl peroxide) are used, and crosslinking is performed in stages in multi-zone furnaces (70–100 °C, 130–200 °C). The second method involves a hydrosilylation reaction with a platinum catalyst. The process can be carried out in a single-zone furnace and at a lower temperature (100–150 °C) [9,10,11]. Silicone PSAs were introduced to the market in the 1960s and have been used in a wide variety of industries [12], e.g., in electronic applications in the manufacturing and assembly of printed circuit boards [13]. Due to the excellent chemical resistance to galvanic solutions, they are utilized in gilding processes [14]. Silicone PSAs have also long been used in healthcare, as transdermal drug delivery systems, and in biomedical devices because these materials are biocompatible (biologically inert, non-toxic, non-irritating, and non-sensitizing) [7]. Research work on new silicone adhesives is mainly focused on their chemical and physical modification of commercially available adhesive binders to increase their thermal resistance [15,16,17,18,19,20,21]. In this work, we propose a new method for obtaining pressure-sensitive silicone adhesives, i.e., a solution-free UV-initiated telomerization process, which is one-step, fast, and energy-efficient (it uses LEDs as a UV source). Telomerization (from Greek telos—end, meros—part) is a radical or ionic chain reaction of a monomer (also called taxogen, M) with a reactive chemical compound (called telogen, YZ). The telomerization reaction results in the formation of telechelic oligomers or polymers (Y-(M)n-Z). Telogens (YZ) can be divided into three main groups: (i) halogen derivatives of hydrocarbons; (ii) compounds containing carbon, hydrogen, sulfur, and oxygen; and (iii) compounds containing a central atom other than carbon [22,23,24]. The latter includes little-known organosilicon telogens. Almost all silicon telogens undergo Si-H bond cleavage. Silicon telegens are characterized by a low chain transfer constant, and halogen substituents affect their activity. The following order of reactivity is usually observed: Cl_3_SiH > CH_3_Br_2_SiH > CH_3_Cl_2_SiH ≫ (RO)_3_SiH > R_3_SiH [25]. There are no previous literature reports on the use of a silicon telogen for photochemically induced telomerization other than our work [6].

In the technology of pressure-sensitive adhesives, the use of adhesion promoters is rather limited to pro-adhesive resins in the formulation of adhesive compositions. On the other hand, primers/activators are used in the PSA technology to modify the surface before the application of the adhesive. The action of these compounds is to improve the wettability of the substrate with a liquid adhesive composition, creating chemical bonds between a film-forming substance (an adhesive binder) and the substrate or creating adhesive–substrate interlayers. The market offers a large number of different chemical varieties of adhesion promoters, including silanes, silicones, titanates, zirconates, hydroxyorganometallic compounds, phosphates, chlorinated polyolefins, modified rosin, and sucrose, as well as naphthenates [26,27]. It is generally assumed that the mode of action of the adhesion promoters is based partly on the formation of chemical bonds with the substrate surface and the binder, and partly on an increase in the wettability of the surface or on the formation of an interlayer between the substrate and binder [28,29,30,31].

The purpose of the presented research was to determine the effect of the type and amount of selected commercial adhesion promoters on the thermal and mechanical properties of a new type of pressure-sensitive adhesives, i.e., silicone–acrylate PSAs (Si-PSAs). It is noteworthy that the new adhesives were obtained exclusively from a silicone–acrylate telomer syrup (Si-AS; prepared via an eco-friendly UV-initiated telomerization process) and adhesion promoter without using proadhesive resins, additional crosslinking agents or fillers increasing thermal stability. Moreover, the crosslinking process relied on a photochemically induced reaction between the photoreactive silicone–acrylate telomer chains. The physicochemical properties of Si-AS and the kinetics of its UV-crosslinking process in the presence of adhesion promoters were studied in detail.

## 2. Materials and Methods

The silicon–acrylate telomer syrup (Si-AS) was prepared using the following components:

- n-butyl acrylate (BA; BASF, Ludwigshafen, Germany);
- acrylic acid (AA; BASF, Ludwigshafen, Germany);
- methyl methacrylate (MMA; BASF, Ludwigshafen, Germany);
- 4-acrylooxybenzophenone (ABP; Chemitec, Scandiccy, Italy);
- ethyl(2,4,6-trimethylbenzoyl)-phenyl phosphinate (APO; Omnirad TPOL, IGM Resins, Waalwijk, The Netherlands);
- triethylsilane (TES; Merck, Warsaw, Poland).

The adhesive compositions were prepared using the telomer syrup and selected adhesion promoters characterized in Table 1.

### 2.1. Silicone–Acrylate Telomer Syrup Preparation and Characterization

**Figure 1:**
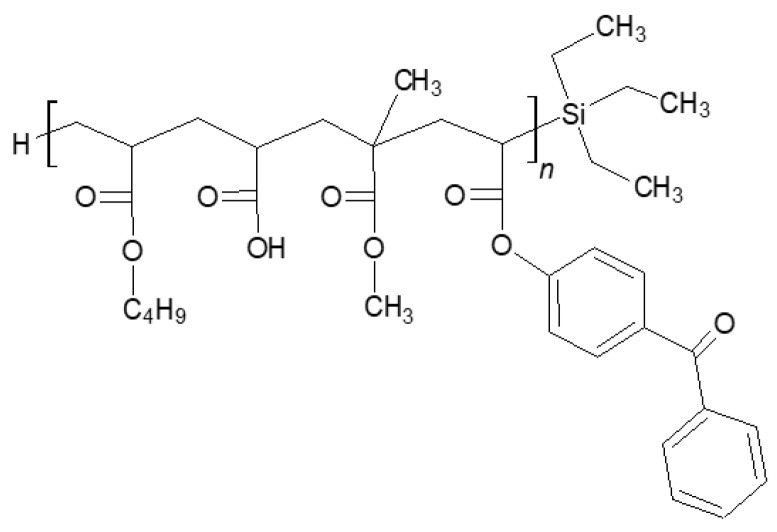
Structure of the silicone–acrylate telomer.

The synthesis and characterization of Si-AS were described earlier [6]. It is a solution of acrylate oligomers containing covalently bonded Si atoms from triethylsilane (a silicone–acrylate telomer, Si-AT) in unreacted monomers (remaining after the telomerization process). The UV-initiated telomerization process (UV-telomerization) was carried out in the presence of the inert gas (Ar) at 20 °C for 30 min in a glass reactor (250 mL) equipped with a mechanical stirrer, thermocouple, and a cooler. The UV-LED stripe (390 ± 5 nm; MEiSSA, Warsaw, Poland) was used as a UV light source. The UV irradiation inside the reactor (5 mW/cm^2^) was controlled using the SL2W UV-radiometer (UV-Design, Brachttal, Germany). A mixture of the reagents containing 86.5 wt. parts of BA, 7.5 wt. parts of AA, 5 wt. parts of MMA, 1 wt. part of ABP, and additionally 10 wt. parts of TES and 0.075 wt. part of APO. The chemical structure of Si-AT is shown in Figure 1. The dynamic viscosity of Si-AS was measured at 23 °C using the DV-II Pro Extra viscometer (spindle #7, 50 rpm; Brookfield, New York, NY, USA). The solids content (SC) of the prepared syrup was determined using the MA 50/1 moisture analyzer (Radwag, Radom, Poland); a sample (ca. 2 g) was heated in an aluminum pan at 105 °C for 4 h. ^1^H and ^29^Si spectroscopic measurements were performed by means of the Bruker DPX 400 Avance III HD spectrometer operating at 400.2 and 100.6 MHz, respectively. For NMR analyses, the MestReNova 12.0.3 software was used. Quantitative analyses were performed using the internal standard (1,3-dinitrobenzene). Differential scanning calorimetry (DSC 250, TA Instruments, New Castle, DE, USA) was used for the determination of the glass transition temperature (T_g_) of Si-AT. A sample (ca. 10 mg) was analyzed in a hermetic aluminum pan from −90 to 200 °C (the heating rate of 10 °C/min).

### 2.2. Pressure-Sensitive Adhesives Preparation and Characterization

Si-PSAs were compounded using Si-AS and the adhesion promoters (0.75; 1 or 1.5 wt. parts per 100 wt. parts of Si-AS). In the case of the TR58 promoter, the addition was 5, 7.5, and 10 wt. parts (according to the manufacturer’s recommendations). The components were mixed using the high-speed mechanical mixer (T10 Basic Ultra-Turrax, IKA, Königswinter, Germany); the prepared mixtures were applied onto a polyester foil and UV-irradiated using the medium-pressure mercury lamp (UV-ABC; Hönle UV-Technology, Gräfelfing, Germany). The UV exposition was controlled with the radiometer (Dynachem 500; Dynachem Corp., Westville, IL, USA). The UV doses reached 1, 2, 3, or 4 J/cm^2^. The base weight of the adhesive layers was ca. 60 g/m^2^.

The kinetics studies of the UV-crosslinking process of the adhesive composition were realized at 25 °C by the photo-DSC method (the differential scanning calorimeter with UV equipment; Q100, TA Instruments, New Castle, DE, USA). During the measurements, a sample (5 mg) was UV-irradiated (250–500 nm) with an intensity of 0.5 W/cm^2^ in the N_2_ atmosphere.

Self-adhesive properties of the UV-crosslinked Si-PSAs were tested according to Association des Fabricants Européens de RubansAuto-Adhésifs (AFERA) standards, i.e., AFERA 5001 (adhesion to a steel substrate, glass, PMMA and PE), AFERA 5015 (tack), and AFERA 5012 (cohesion at 20 °C). These parameters were evaluated using three samples of each adhesive tape using the Z010 machine (Zwick/Roell, Ulm, Germany). Generally, adhesion is defined as a force value required to remove a pressure-sensitive material from a substrate (stainless-steel plate or other); the process is realized at an angle of 180° at a removal speed of 300 mm/min. Tack is characterized as a force value required for the separation of a stainless-steel plate and an adhesive tape applied under low pressure (contact time of 0.5 s). Cohesion (i.e., static shear adhesion) describes the time needed to shear off an adhesive tape sample (under a load of 1 kg) from a defined steel surface. Differential scanning calorimetry (DSC 250) was used for the determination of the glass transition temperature (T_g_) of UV-crosslinked Si-PSAs. A sample (ca. 10 mg) was analyzed using a hermetic aluminum pan from −90 to 200 °C (the heating rate of 10 °C/min). Two DSC measurements for each composition were carried out. The contact angles for water/Si-PSA systems were measured with the EO 300A goniometer (Surface & Electro-Optics, Suwon-si, Republic of Korea). A single drop of distilled water was placed on the Si-PSA surface; the contact angle was observed after 5 s using a high-speed camera (1000 frames/s).

The thermal stability of the UV-crosslinked Si-PSAs (in the N_2_ atmosphere) was measured by using the TGA (TG500 IR, TA Instrument, New Castle, DE, USA). A sample (ca. 10 mg) was heated in an aluminum pan at the temperature range of 25–900 °C (10 °C/min). The initial decomposition temperature, at which 5% of the material evaporated (T_5%_), as well as the temperature of the loss of 50% of the sample mass (T_50%_) were determined.

## 3. Results

### 3.1. The Physicochemical Properties of the Silicone–Acrylate Telomer Syrup

**Figure 2:**
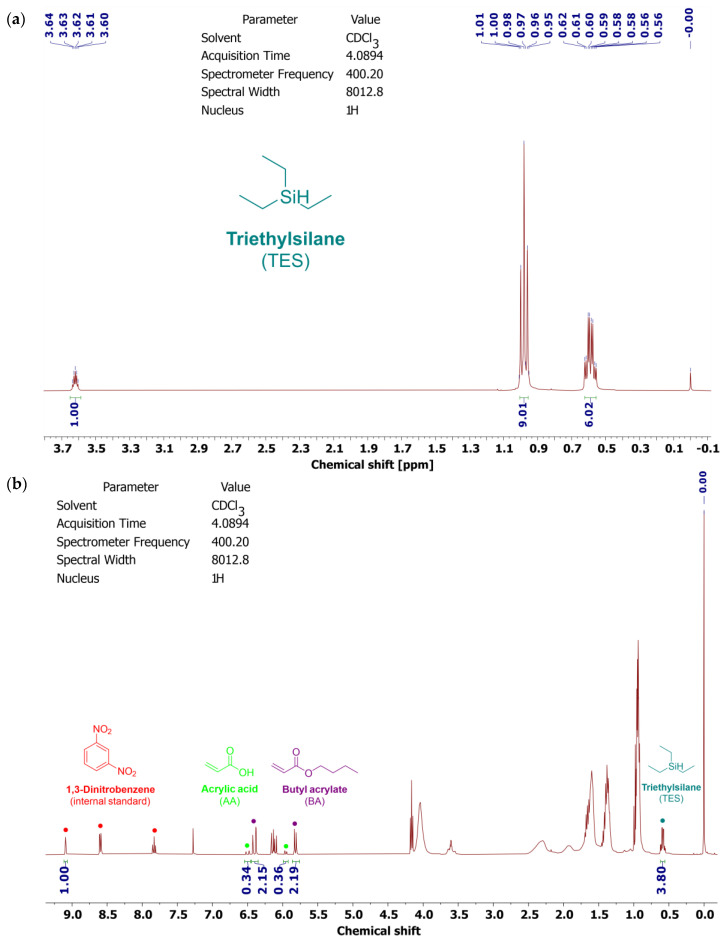
^1^H NMR spectra of the telogen (triethylsilane) (**a**) and Si-AS (**b**).

Selected physicochemical properties of the prepared telomer syrup are presented in Table 2. Additionally, ^1^H NMR spectra for the Si-telogen and Si-AS are shown in Figure 2.

**Figure 3:**
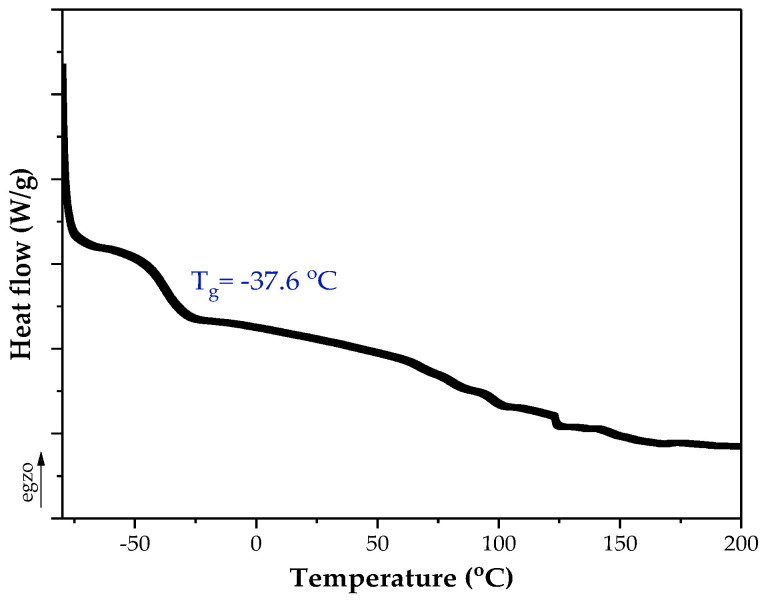
DSC thermograph of the silicone–acrylate telomer.

As revealed, Si-AS was characterized by a high solids content (79%), which correlated with the value of the total monomers conversion from the NMR tests. The NMR studies of the syrup proved that silicon atoms are covalently bound to the polyacrylate structure (TES conversion was 59%). In Si-AS, which is a post-reaction mixture, silicone–acrylate telomer chains (81 wt.%) as well as unreacted monomers (BA, AA) and TES were present. A type II photoinitiator (ABP) was completely incorporated, indicating that the telomeric chains were photoreactive. Furthermore, the DSC analysis (Figure 3) showed that the silicone–acrylate telomer (i.e., the syrup without unreacted monomers) had a low glass transition temperature (−37 °C) desirable for pressure-sensitive adhesives.

### 3.2. Influence of the Adhesion Promoters on the UV-Crosslinking Process of Si-AS

**Figure 4:**
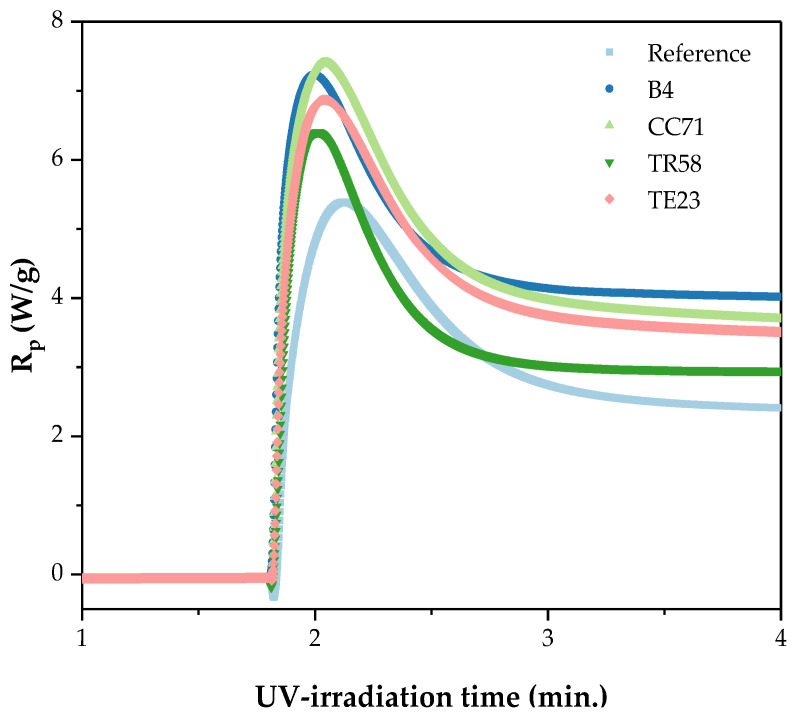
Kinetic curves of the UV-crosslinking process of Si-AS without (the reference sample) and with the different adhesion promoters (I_0_ = 0.5 W/m^2^; 250–500 nm).

**Figure 5:**
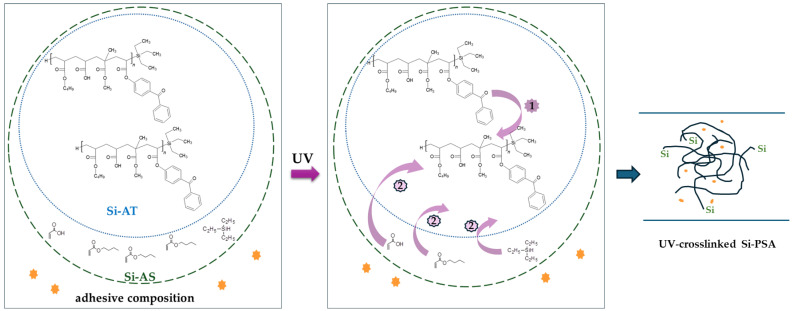
Proposed mechanism of the UV-crosslinking process of Si-AS (_-_adhesion promoter).

The study aimed to determine the effect of physical modification of the silicone–acrylate telomer syrup (using the selected commercial adhesion promoters) on thermal stability as well as mechanical properties of the new pressure-sensitive adhesives. The key stage of the research was to optimize the composition of the adhesive formulations (telomeric syrup + adhesion promoter) and their UV-crosslinking conditions to obtain adhesives with desired cohesion (72 h), high adhesion to steel, and tack. First, the effect of the adhesion promoters on the UV-crosslinking process of the adhesive composition was studied. In this test, the same addition (1 wt. part/100 wt. parts of Si-AS) of different adhesion promoters was investigated. The results of the photo-DSC analysis are shown in Figure 4 and Table 3. Previously, we suggested that crosslinking of a telomer syrup involves radical polymerization of the remaining acrylate monomers under the influence of radicals formed by hydrogen abstraction from methyl methacrylate molecules (unreacted MMA in the telomer syrup) by a benzophenone moiety (from ABP incorporated into the telomer structure) [6]. However, as NMR studies have shown, MMA is completely incorporated into the telomer chain structure in the prepared syrup (Table 2), and thus the hydrogen abstraction can occur from the primary carbon atom (the CH_3_ side group) of the MMA molecules incorporated into the telomer structure (Figure 5). It is generally known that type II photoinitiators (such as ABP) need a co-initiator to conduct a UV-crosslinking process (e.g., tertiary amines) [32].

From our unpublished studies, we know that a self-crosslinking process of a silicon–acrylate telomer syrup (without photoinitiator) does not occur in MMA-free systems (BA/AA/ABP/TES). The UV-crosslinking ability of the investigated Si-AS system was confirmed by the photo-DSC studies (Figure 4); in the case of the unmodified syrup, the released heat amount (ΔH) was 133 J/g, and the maximum reaction rate was recorded after ca. 22 s of the UV-exposure. The research showed that all the adhesion promoters increase the UV-crosslinking rate of the Si-AS syrup—the best result was revealed for the system with the hydroxymetal-organic compound (CC7). This is evidenced by the highest reaction heat (138 J/g) and the shortest time to reach R_p_^max^ (only 13.2 s). It is known that a photopolymerization process occurs faster in the presence of monomers with hydroxyl groups and/or heteroatoms [33,34]. Nevertheless, the shortest time to achieve the maximum reaction rate was noted for the sample modified with TR58 (11.4 s). It is affected by the presence of the UV-crosslinkable unsaturated compound (HDDA) in this modifier. However, this resulted in a small amount of the released heat (88 J/g), and thus it can be concluded that the degree of crosslinking was also lower for this sample (as is typical for multifunctional systems). Generally, the photopolymerization rate (represented by the Rp values [33]) decreases in the following order of the applied adhesion promoters: CC71 > B4 > TE23 > TR58 > no adhesion promoter.

**Figure 6:**
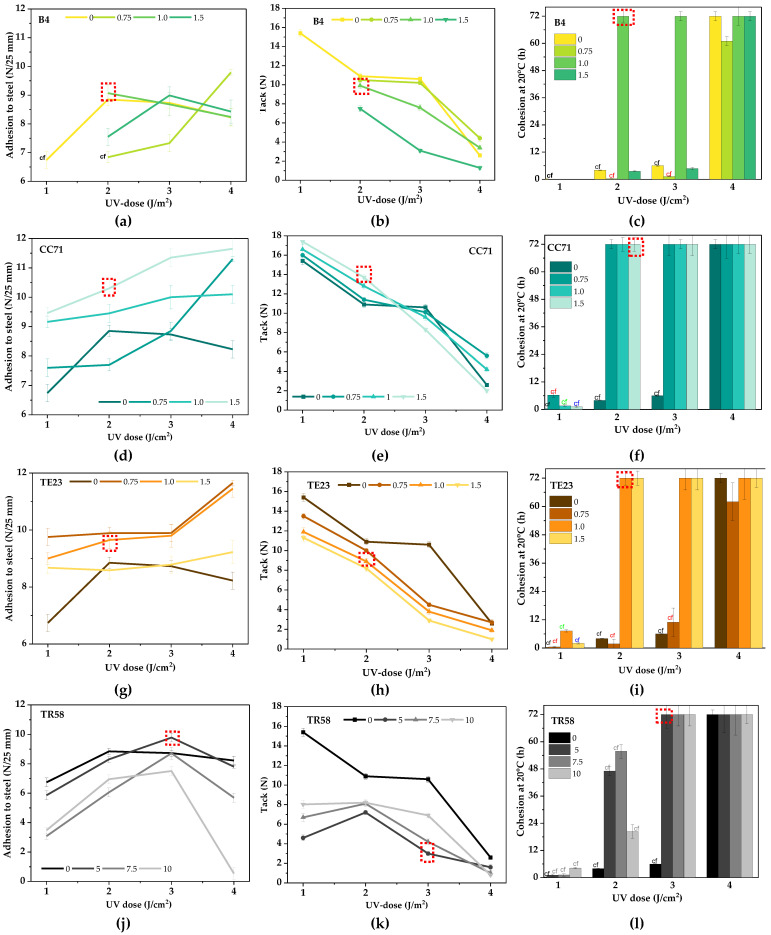
Adhesion to steel (**a**,**d**,**g**,**j**), tack (**b**,**e**,**h**,**k**), and cohesion at 20 °C (**c**,**f**,**i**,**l**) of Si-PSAs with different adhesion promoters (cf, cohesive failure).

The positive effect of the adhesion promoters on the UV-crosslinking rate was also confirmed by the results of adhesion to steel, tack, and cohesion at 20 °C of Si-AS-based pressure-sensitive adhesives (Figure 6). In this case, different doses of the modifiers (the TR58 concentration was deliberately higher) and the UV light were tested. The study showed that the reference sample (Si-PSA/0) required a higher UV dose (4 J/cm^2^) for sufficient crosslinking (no cohesive failure of the adhesive film). Under these conditions, the desired cohesion at 20 °C was obtained (72 h); however, the tack and adhesion to steel were relatively low (3 N and 8.3 N/25 mm, respectively). The result of the latter parameter was satisfactory, but average. The best adhesion to steel, tack, and cohesion for Si-PSAs with adhesion promoters are marked in red in Figure 6. In the case of the samples with the B4 modifier (a copolymer with -OH and -COOH functional groups), it was found that adhesion is a function of promoter concentration and UV dose (there are maxima of the parameter, Figure 6a). The adhesion value was more affected by the cross-linking density of the adhesive film (in general, an adhesion decrement was observed at higher UV doses) than by the adhesion promoter concentration. In contrast, the tack of Si-PSA/B4 decreased with increasing UV dose and the B4 modifier concentration, but the values were lower than for the reference sample. Nevertheless, the highest cohesion values were recorded for the adhesive films crosslinked with the highest UV dose. In the case of the Si-PSA/B4 systems, an improvement of adhesion to steel (+18%) and tack (+296% vs. Si-PSA/0) as well as good cohesion (72 h) were registered for a sample containing 1 wt. part of B4 and crosslinked with a UV dose of 2 J/cm^2^ (Si-PSA/B4 (1/2)). On the other hand, using the hydroxymetal-organic compound (Si-PSA/CC71), the adhesion to steel increased with the increasing concentration of this promoter and the UV dose. There was no destruction of the adhesive films (the samples were already crosslinked properly at the lowest UV dose). These results correlate with the above-described photo-DSC study—it revealed that CC71 has the strongest influence on increasing the UV-crosslinking rate. In general, the adhesion was higher than for the reference sample. The hydroxymetallic compounds contain a hydroxyl group (reacting with the substrate), a metal atom, and a long organic substituent (with amino groups) that interact with the adhesive binder. For this reason, the more CC71 in the system, the better the adhesion (more interactions at the adhesive–steel interface) because the adhesive is enriched by hydroxyl groups from CC71.

On the contrary, the tack of Si-PSA/CC71 decreased with increasing UV dose. This is caused by an increment of the cross-linking density of the adhesive film (and thus tack is reduced as well). In addition, the tack test involves a very short contact between the adhesive film and the substrate (a few seconds) and it was not possible to form hydrogen bonds between the adhesive layer (rich in -OH groups from CC71) and the steel surface. In the case of adhesion tests, the contact time between the layers (adhesive and steel) was 20 min, and thus the cross-linking density was not as important as during the tack measurements. Already at the UV dose of 2 J/cm^2^, good-quality adhesive films could be obtained (cohesion at 20 °C was 72 h). Improved adhesion to steel (+28%) and tack (+488%) as well as a high cohesion (72 h) were registered for the adhesive film with 1.5 wt. part of CC71 and crosslinked with the UV dose of 2 J/cm^2^ (Si-PSA/CC71 (1.5/2)).

The TE23 modifier is a quaternary ammonium salt, and this solvent-free, antistatic additive increases the electrical conductivity of organic coatings. Despite this, the positive effects of this compound in the tested PSAs have been observed. The adhesion to steels of Si-PSA/TE23 was significantly higher in relation to the reference sample. It may result from its migration into the top layer of Si-PSA and the creation of a hygroscopic layer that attracts water (from the steel surface). Thus, adhesion to steel increases with decreasing concentration of this additive and the UV light dose. It is noteworthy that the tack was reduced by the higher radiation doses and—generally—its values were lower than for the reference sample. This effect is related to the high cross-linking density of the adhesive film; it was shown that TE23 increases the UV-crosslinking rate of the Si-AS and, additionally, the higher dose of this additive, the lower viscosity of the adhesive composition (hence the UV-crosslinking process occurs even more easily). The most interesting properties of TE23-based systems, i.e., improved adhesion to steel (+20%) and tack (+394%) (cohesion of 72 h), were registered for the adhesive film containing 1 wt. part of TE23 and crosslinked by 2 J/cm^2^ (Si-PSA/TE23 (1/2)).

In the case of systems with chlorinated polyolefin TR58, adhesion to steel was lower than for the reference samples (regardless of the promoter concentration and the UV dose), except for the sample with the lowest concentration of this additive (5 wt. parts). Moreover, adhesion of the TR58-based Si-PSAs increased with increasing radiation dose, but only in the range of 1–3 J/cm^2^ (at higher doses, it was significantly reduced). The more TR58 in the system, the lower the adhesion values (due to the increment of cross-linking density). On the other hand, the tack values decreased at higher doses of UV radiation. At the dose of 3 J/cm^2^, regardless of the adhesion promoter concentration, good-quality adhesive films with TR58 could be obtained (cohesion of 72 h). Generally, CPO-type compounds are rarely used as formulation additives, but more often as primers. The less beneficial effect of this adhesion promoter (than the others) may result from the presence of HDDA in TR58 (it increases crosslinking density and lowers adhesion, but improves the cohesion parameter) and the fact that CPOs generally tend to release HCl under certain conditions. Considering the Si-PSAs with that modifier, the highest adhesion to steel (+22.5%) and tack (+152%) (at the cohesion of 72 h) was registered for the adhesive film containing 5 wt. parts of TR58 (3 J/cm^2^) (Si-PSA/TR58 (5/3)). A summary of the best results of adhesion properties of the prepared Si-PSAs is presented in Table 4.

**Figure 7:**
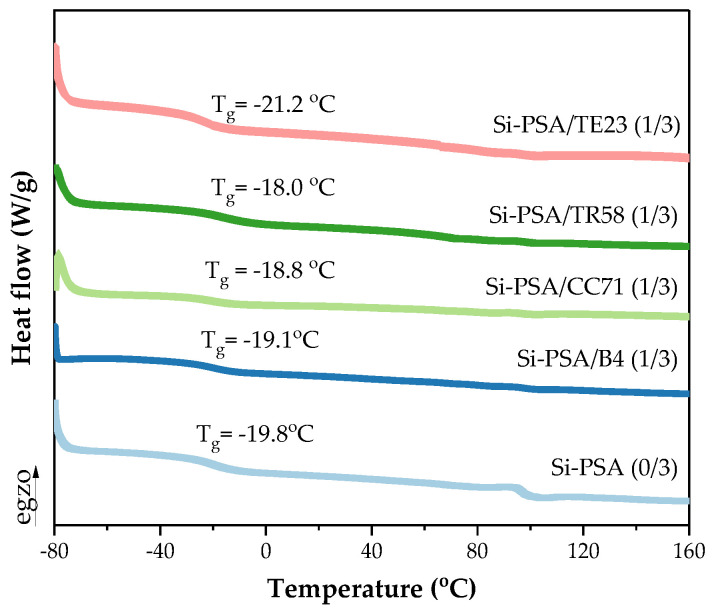
DSC thermograph for Si-PSAs with different adhesion promoters (1 wt. part) crosslinked with the UV dose of 3 J/cm^2^.

The DSC thermal analysis was performed for the UV-crosslinked (3 J/cm^2^) adhesive films (containing 1 wt. part of the adhesion promoters). Generally, the results (Figure 7) did not show significant differences in the T_g_ values of the tested Si-PSAs ((−18.0)–(−19.8) °C); only the system with the quaternary ammonium salt (TE23) slightly stood out (−21.2 °C). Thus, it can be concluded that the noted improvement (or deterioration) of the adhesive properties of Si-PSAs does not depend on their glass transition temperature values.

**Figure 8:**
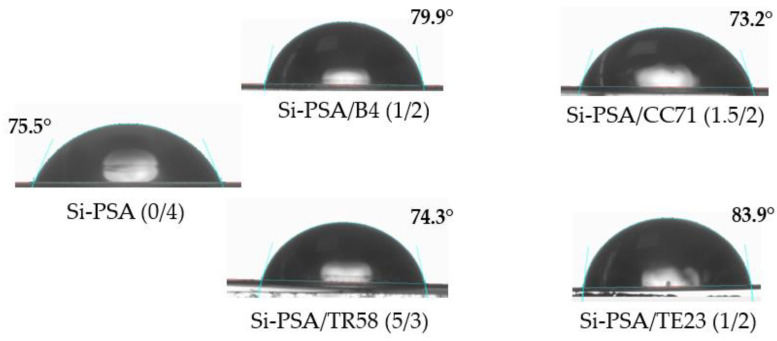
Images from measurements of water contact angle for the selected Si-PSA systems.

The results of the Si-PSAs wettability test are shown in Figure 8. Compared to the reference sample (Si-PSA (0/4)), the samples with the TE23 or B4 modifiers showed a more hydrophobic character (83.9° and 79.9°, respectively). On the other hand, the addition of the hydroxymetal-organic promoter (CC71) or the chlorinated polyolefin (TR58) resulted in a lower hydrophobicity of the film. The values of the Si-PSA/water wetting angle are as follows for the tested adhesion promoters: CC71 < TR58 < no adhesion promoter < B4 < TE23.

In the next step, the adhesion of the selected Si-PSA systems to various substrates (i.e., glass, PMMA, PE) was studied. The results are summarized in Table 5. Generally, the water wetting angle values for all the selected Si-PSAs systems were lower than 90°, which indicated their hydrophilic nature. It was confirmed by their adhesion to PE—all the samples were characterized by values lower than 1 N/25 mm. Interestingly, the most limited adhesion (0.03 N/25 mm) was registered for the TR58-based sample (this adhesion promoter is mainly dedicated to PE and PP substrates).

That type of the modified Si-PSA reached the lowest adhesion to the other substrates as well. Nevertheless, in the case of the glass and PMMA panels, the modified Si-PSAs films exhibited markedly higher adhesion (8.9–15.0 N/25 mm and 5.1–12.5 N/25 mm, respectively) in comparison with the reference sample (6.1 and 2.7 N/25 mm). Generally, the most favorable influence on adhesion to glass, PMMA, and PE was recorded for the Si-PSA system with B4 (followed by CC71 and TE23).

**Figure 9:**
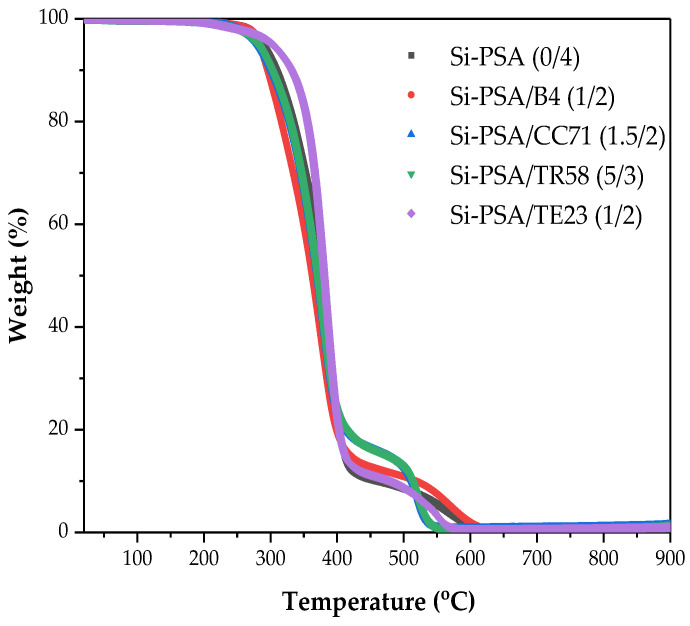
TGA curves for the selected Si-PSA systems.

Due to the chemical structure of prepared Si-PSAs, including the presence of silicon atoms, the most interesting study concerned their thermogravimetric tests illustrating their thermal stability. The results are presented in Figure 9 and Table 6. As can be seen, the temperature at the specific weight loss of samples directly depends on the type of adhesion promoter (and crosslinking degree of the system). It was found that the thermal stability of the modified Si-PSAs tapes increased as follows: CC71 < TR58< B4 < no adhesion promoter < TE23. Interestingly, the reference sample exhibited almost the same thermal stability (comparing decomposition temperature values at 5% weight loss) as the commercial silicone adhesive Dowsil Q2-7566 (287 °C vs. 289 °C) [35]. On the other hand, Si-PSA/TE23 (1/2) modified by the quaternary ammonium salt was significantly more thermally stable (T_5%_ = 302 °C) than the commercial product. It turned out that the other tested adhesion promoters negatively affected the thermal stability of the telomeric-syrup-based Si-PSA. This probably resulted from their influence on the photocrosslinking process (during the Si-PSA film preparation), mainly on the density of the created polymer network. Arguably, the composition of the applied adhesion promoters may affect the thermal stability of the Si-PSA as well. It is known that quaternary ammonium salt is often stable above 300 °C [36]; however, the concentration of the TE23 additive in the tested sample was relatively too low to directly increase the values of the T_5%_ and T_50%_ parameters.

## 4. Conclusions

Taking into consideration the test results of the silicone–acrylate telomer syrup (Si-AS) modified with selected adhesion promoters as well as the UV-curable pressure-sensitive adhesives (Si-PSAs based on Si-AS), the following conclusions can be drawn:

- Hydroxymetal-organic compounds (CC71) seem to be the best adhesion promoters for Si-PSAs. Significant increments of tack (+488%) and adhesion to steel (+28%) were observed after the addition of this modifier (1.5 wt. parts/100 wt. parts of Si-AS) and photocrosslinking using the relatively low UV dose (2 J/cm^2^). The observed improvements resulted from the increased UV-crosslinking rate of the Si-AS/CC71 system (vs. Si-AS). Very high adhesion of this tape to PMMA was also observed;
- The quaternary ammonium salt-based additive (TE23) markedly increased tack (+240%) and adhesion to steel (+20%) of the Si-PSAs as well. The influence was observed at the low concentration of TE23 (1 wt. part) and the low UV dose (2 J/cm^2^). Moreover, the Si-PSA/TE23 system shows the highest thermal stability (T_5%_ = 302 °C and T_5o%_ = 379 °C) and high adhesion to PMMA in relation to the other systems and the unmodified sample (287 °C and 376 °C, respectively);
- The chlorinated polyolefin (CC71) (5 wt. parts) and the UV dose of 3 J/cm^2^, nevertheless, provide high adhesion to glass and PMMA, but low adhesion to PE;
- An influence of the organic copolymer with hydroxyl and carboxyl groups (B4) on adhesion to the steel of Si-PSAs is relatively limited; however, values of adhesion to glass, PMMA, or PE recorded for the Si-PSA/B4 system were almost the highest in comparison with the other compositions.

In conclusion, Si-PSAs based on the silicone–acrylate telomer syrup and the selected adhesion promoters are characterized by (i) high thermal stability (up to 302 °C), (ii) excellent adhesion to glass, PMMA, and steel, and (iii) limited adhesion to PE.

## Figures and Tables

**Table 1:** Characteristic of adhesion promoter.

Type	Name (Manufacturer) Symbol	Features
Organic copolymer with polar groups	Byk 4510 (Byk-Chemie, Wesel, Germany) B4	Solution of a hydroxy-functional copolymer with acidic groups Acid value: 30 mg KOH/g
Hydroxymetal-organic compound	Chartwell C-515.71HR (Old York, NY) CC71	Amino-functional type Metal content: 7.3–7.9 wt.%
Quaternary ammonium salt	Tego ADDID 230 (Evonik Ind., Essen, Germany) TE23	Active matter content: 100%
Chlorinated polyolefin (CPO)	Trapylen 5800 UV (Tramaco, Tornesch, Germany) TR58	Solution in HDDA; Cl content: 42 wt.%

**Table 2:** Main features of Si-AS.

Solids content (%)	Viscosity (Pa∙s)	Conversion of the monomers (%) *					Total conversion (%) *
		BA	AA	MMA	ABP	TES	
79	12	82.22	81.41	100	100	59.28	81
		Content of free monomers (wt.%) *					Telomer content (wt.%)
		73.7	6.7	0	0	19.6	81

* based on ^1^H NMR studies.

**Table 3:** Characteristic parameters of the UV-crosslinking process of Si-AS modified with the adhesion promoters (I_0_ = 0.5 W/m^2^; 250–500 nm).

Sample	t_Rp_^max^ (s)	ΔH (J/g)
Si-AS	22.8	133
Si-AS/B4	12.0	91
Si-AS/CC71	13.2	138
Si-AS/TE23	13.2	120
Si-AS/TR58	11.4	88

**Table 4:** Summary of the adhesive properties of the selected Si-PSA systems.

Sample	Adhesion to Steel (N/25 mm)	Tack (N)	Cohesion at 20 °C (h)
Si-PSA (0/4)	8.0 ± 0.5	2.5 ± 0.4	72
Si-PAS/B4 (1/2)	9.5 ± 0.6	9.9 ± 0.6	72
Si-AS/CC71 (1.5/2)	10.3 ± 0.4	14.7 ± 0.5	72
Si-PAS/TE23 (1/2)	9.6 ± 0.3	9.8 ± 0.3	72
Si-AS/TR58 (5/3)	9.8 ± 0.2	3.8 ± 0.3	72

**Table 5:** Adhesion of the selected Si-PSA systems to various substrates.

Sample	Adhesion (N/25 mm^2^)		
	Glass	PMMA	PE
Si-PSA (0/4)	6.1 ± 0.4	2.7 ± 0.3	0.06 ± 0.02
Si-PSA/B4 (1/2)	15.0 ± 0.3	12.1 ± 0.4	0.41 ± 0.02
Si-PSA/CC71 (1.5/2)	13.9 ± 0.4	12.5 ± 0.3	0.15 ± 0.01
Si-PSA/TR58 (5/3)	8.9 ± 0.4	5.1 ± 0.6	0.03 ± 0.01
Si-PSA/TE23 (1/2)	13.5 ± 0.4	12.5 ± 0.4	0.19 ± 0.01

**Table 6:** Thermal stability parameters for the selected Si-PSA systems.

Samples	T_5%_ (°C)	T_50%_ (°C)
Si-PSA (0/4)	287	376
Si-PSA/B4 (1/2)	283	362
Si-PSA/CC71 (1.5/2)	280	370
Si-PSA/TR58 (5/3)	280	373
Si-PSA/TE23 (1/2)	302	379

## Data Availability

The original contributions presented in the study are included in the article, further inquiries can be directed to the corresponding author.
